# (1*R*,4′*S*)-4-(*tert*-Butyl­dimethyl­silan­oxy)-1-[2,2-dimethyl-3-(*p*-tolyl­sulfon­yl)-1,3-oxazolidin-4-yl]but-2-yn-1-ol

**DOI:** 10.1107/S1600536808014906

**Published:** 2008-05-30

**Authors:** Jörg Erdsack, Markus Schürmann, Hans Preut, Norbert Krause

**Affiliations:** a Fachbereich Chemie, Technische Universität Dortmund, Otto-Hahn-Strasse 6, 44221 Dortmund, Germany

## Abstract

The chiral title compound, C_22_H_35_NO_5_SSi, is a precursor of novel furan­omycin derivatives. It crystallizes with two molecules in the asymmetric unit; these show different conformations of the silyl substitutent, as indicated by the Si—O—C—C torsion angles of 41.4 (7) and −84.5 (5)° in the two mol­ecules. The *anti* configuration of the adjacent stereogenic centers is consistent with the Felkin–Anh model. Each of the two crystallographically independent mol­ecules is connected with a neighbouring mol­ecule of the same type *via* two symmetry-equivalent O—H⋯O hydrogen bonds.

## Related literature

For related literature, see: Anh & Eisenstein (1977 ▶); Chérest *et al.* (1968 ▶); Deutsch *et al.* (2008 ▶); Erdsack & Krause (2008 ▶); Garner & Park (1987 ▶); Hoffmann-Röder & Krause (2001 ▶); Kim & Rhee (2000 ▶).
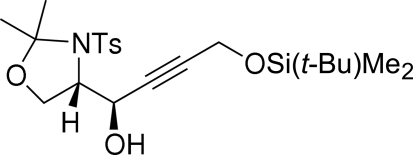


## Experimental

### 

#### Crystal data

C_22_H_35_NO_5_SSi
*M* *_r_* = 453.66
Monoclinic, 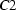

*a* = 26.283 (5) Å
*b* = 11.335 (2) Å
*c* = 19.219 (4) Å
β = 121.40 (3)°
*V* = 4887 (2) Å^3^
*Z* = 8
Mo *K*α radiation
μ = 0.21 mm^−1^
*T* = 173 (1) K
0.10 × 0.08 × 0.01 mm

#### Data collection

Nonius KappaCCD diffractometer
Absorption correction: none
8353 measured reflections
8353 independent reflections
3487 reflections with *I* > 2σ(*I*)
*R*_int_ = 0.035

#### Refinement

*R*[*F* ^2^ > 2σ(*F* ^2^)] = 0.035
*wR*(*F* ^2^) = 0.087
*S* = 1.03
8353 reflections
559 parameters
1 restraint
H-atom parameters constrained
Δρ_max_ = 0.18 e Å^−3^
Δρ_min_ = −0.20 e Å^−3^
Absolute structure: Flack (1983 ▶), 3636 Friedel pairs
Flack parameter: −0.09 (7)

### 

Data collection: *COLLECT* (Nonius, 1998 ▶); cell refinement: *DENZO* and *SCALEPACK* (Otwinowski & Minor, 1997 ▶); data reduction: *DENZO* and *SCALEPACK*; program(s) used to solve structure: *SHELXS97* (Sheldrick, 2008 ▶); program(s) used to refine structure: *SHELXL97* (Sheldrick, 2008 ▶); molecular graphics: *SHELXTL-Plus* (Sheldrick, 2008 ▶); software used to prepare material for publication: *SHELXL97* and *PLATON* (Spek, 2003 ▶).

## Supplementary Material

Crystal structure: contains datablocks I, global. DOI: 10.1107/S1600536808014906/hb2724sup1.cif

Structure factors: contains datablocks I. DOI: 10.1107/S1600536808014906/hb2724Isup2.hkl

Additional supplementary materials:  crystallographic information; 3D view; checkCIF report

## Figures and Tables

**Table 1:** Hydrogen-bond geometry (Å, °)

*D*—H⋯*A*	*D*—H	H⋯*A*	*D*⋯*A*	*D*—H⋯*A*
O4—H4⋯O2^i^	0.84	2.01	2.828 (4)	164
O4′—H4′⋯O2′^ii^	0.84	2.06	2.851 (4)	157

Symmetry codes: (i) 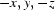; (ii) 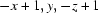.

**Fig. 1.**
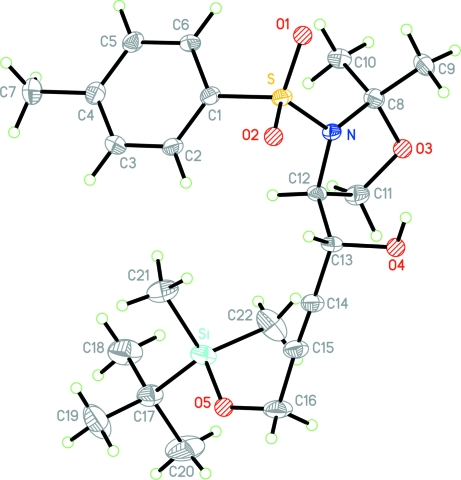
: The molecular structure of molecule 1 in (I) with displacement ellipsoids for the non-H atoms shown at the 30% probability level.

**Fig. 2.**
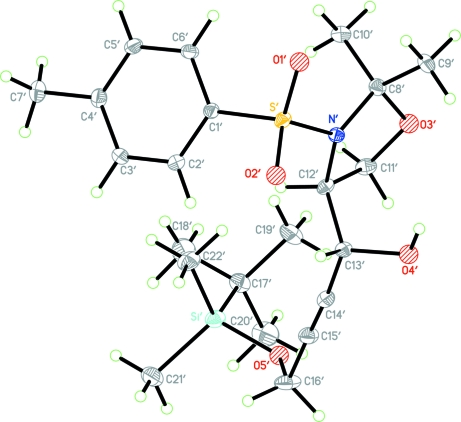
: The molecular structure of molecule 2 in (I) with displacement ellipsoids for the non-H atoms shown at the 30% probability level.

## supplementary crystallographic information

### Comment

The title compound, (I), is a precursor of novel furanomycin derivatives using the gold-catalyzed cyclization of α-hydroxyallenes (Hoffmann-Röder & Krause, 2001; Erdsack & Krause, 2008), which are prepared by S_N_2' substitution of propargylic carbonates with catalytic copper hydride (Deutsch *et al.*,  2008). The compound (I) was synthesized by nucleophilic acetylide addition to the tosyl protected analogue of Garner's aldehyde (Kim & Rhee, 2000; Garner & Park, 1987) under non-chelating conditions. A crystal structure determination of (I) has now been carried out to establish the *anti* configuration of the adjacent stereocenters. Figs. 1 and 2 show that the relative configuration of C12 and C13 in the first molecule and C12' and C13' in the second molecule are as expected.

The compound crystallizes in pairs of molecules, which are different concerning the conformation of the silyl substitutent, as indicated by the Si—O5—C16—C15 and Si'—O5'—C16'—C15' torsion angles of 41.4 (7)° and -84.5 (5)°, respectively.  The torsion angles C17—Si—O5—C16 [155.8 (4)°] and C17'—Si'—O5'—C16' [169.9 (4)°] also differ significantly.  The preferred *gauche* conformation of the substitutents O4 and C11 along the newly formed C12—C13 bond is in accordance with the Felkin-Anh model (Chérest *et al.*,  1968; Anh & Eisenstein, 1977). The configuration of the stereogenic centers in (I) (C12  *S* and C13 *R*) was assigned based on the starting material (*L*-serine).

In the crystal, pairs of symmetry equivalent molecules are linked by pairs of O—H···O hydrogen bonds (Table 1).

### Experimental

In an oven-dried three-necked 250-ml-flask equipped with a magnetic stirring bar and nitrogen inlet, *tert*-butyldimethylprop-2-ynyloxysilane (4.68 g, 27.5 mmol) was dissolved in anhydrous THF (95 ml) under argon. After cooling to 243 K, n-BuLi (2.5 *M* in hexane, 11.0 ml, 27.5 mmol) was added dropwise and stirring was continued for 15 min. Hexamethylphosphoramide (4.78 ml, 27.5 mmol) was added and the solution was cooled to 188 K. After 5 min with stirring, a solution of (*S*)-2,2-dimethyl-3-(toluene-4-sulfonyl)-oxazolidine-4-carbaldehyde (3.9 g, 13.8 mmol) in anhydrous THF (20 ml) was added slowly by syringe on the inner surface of the flask. After 45 min with stirring at 188 K, TLC showed the complete conversion of starting material. After 60 min total time of stirring, the mixture was poured into aq. sat. NH_4_Cl (250 ml). The organic phase was separated and the residue was washed with diethyl ether (3 × 100 ml). The combined organic layers were washed with brine, dried (MgSO_4_), filtered, and the solvent was evaporated. The residue was purified by column chromatography (silica gel, isohexane-EtOAc 85:15 → 7:3) to give the title compound (I) (5.13 g, 82%) as a colourless solid. A small sample was recrystallized from isohexane to give colourless blocks and plates of (I) suitable for X-ray analysis; mp 388 K (isohexane); [α]^21^_D_ -38.6 (c 1.20, CHCl_3_); *R*_f_ = 0.66 (isohexane-EtOAc 7:3); IR (KBr): 3514, 2984, 2952, 2928, 2885, 2857, 1599, 1471, 1461, 1385, 1339, 1254, 1205, 1159, 1090, 1030, 837, 815, 779, 660, 594 cm^-1^; ^1^H NMR (400 MHz, CDCl_3_): δ (p.p.m.) = 7.73 (d, J = 8.2 Hz, 2 H), 7.29 (d, J = 8.1 Hz, 2 H), 4.73 (d, J = 3.9 Hz, 1 H), 4.30 (d, J = 1.4 Hz, 2 H), 4.20 (dd, J = 4.0, 9.3 Hz, 1 H), 3.96 (dd, J = 7.1, 9.2 Hz, 1 H), 3.79 (m, 1 H), 3.07 (d, J = 6.1 Hz, 1 H), 2.40 (s, 3 H), 1.68 (s, 3 H), 1.50 (s, 3 H), 0.87 (s, 9 H), 0.08 (s, 6 H); ^13^C NMR (100 MHz, CDCl_3_): δ (p.p.m.) = 143.8, 136.8, 129.7, 127.5, 99.2, 85.3, 82.0, 65.0, 62.6, 62.5, 51.5, 28.3, 25.7, 24.7, 21.4, 18.2, -5.3; HRMS (ESI): m/*z* [*M* + H]^+^ calcd for C_22_H_36_NO_5_^32^S^28^Si: 454.2078; found 454.2072.

### Refinement

The H atoms were placed in calculated positions, with C—H = 0.95–1.00 and O—H = 0.84 Å and refined as riding, with *U*_iso_= 1.5*U*_eq_ for the methyl groups and *U*_iso_= 1.2*U*_eq_ for the remaining H positions; the methyl groups were allowed to rotate but not to tip to best fit the enectron density.

### Crystal data

**Table:**

C_22_H_35_NO_5_SSi	*F*_000_ = 1952
*M**_r_* = 453.66	*D*_x_ = 1.233 Mg m^−^^3^
Monoclinic, *C*2	Mo *K*α radiation λ = 0.71073 Å
Hall symbol: C 2y	Cell parameters from 20719 reflections
*a* = 26.283 (5) Å	θ = 2.6–25.3º
*b* = 11.335 (2) Å	µ = 0.21 mm^−^^1^
*c* = 19.219 (4) Å	*T* = 173 (1) K
β = 121.40 (3)º	Plate, colourless
*V* = 4887 (2) Å^3^	0.10 × 0.08 × 0.01 mm
*Z* = 8	

### Data collection

**Table:**

Nonius KappaCCD diffractometer	8353 independent reflections
Radiation source: fine-focus sealed tube	3487 reflections with *I* > 2σ(*I*)
Monochromator: graphite	*R*_int_ = 0.035
Detector resolution: 19 vertical, 18 horizontal pixels mm^-1^	θ_max_ = 25.3º
*T* = 173(2) K	θ_min_ = 2.6º
286 frames via ω–rotation (Δω=1°) and two times 120 s per frame (3 sets at different κ–angles) scans	*h* = −31→31
Absorption correction: none	*k* = −12→13
8353 measured reflections	*l* = −23→22

### Refinement

**Table:**

Refinement on *F*^2^	Hydrogen site location: inferred from neighbouring sites
Least-squares matrix: full	H-atom parameters constrained
*R*[*F*^2^ > 2σ(*F*^2^)] = 0.035	[1.0exp(6.50(sinθ/λ)^2^)]/[σ^2^(*F*_o_^2^)]
*wR*(*F*^2^) = 0.087	(Δ/σ)_max_ = 0.004
*S* = 1.03	Δρ_max_ = 0.18 e Å^−^^3^
8353 reflections	Δρ_min_ = −0.20 e Å^−^^3^
559 parameters	Extinction correction: none
1 restraint	Absolute structure: Flack (1983), 3636 Friedel pairs
Primary atom site location: structure-invariant direct methods	Flack parameter: −0.09 (7)
Secondary atom site location: difference Fourier map	

### Special details

**Table:**

Geometry. All e.s.d.'s (except the e.s.d. in the dihedral angle between two l.s. planes) are estimated using the full covariance matrix. The cell e.s.d.'s are taken into account individually in the estimation of e.s.d.'s in distances, angles and torsion angles; correlations between e.s.d.'s in cell parameters are only used when they are defined by crystal symmetry. An approximate (isotropic) treatment of cell e.s.d.'s is used for estimating e.s.d.'s involving l.s. planes.
Refinement. Refinement of *F*^2^ against ALL reflections. The weighted *R*-factor *wR* and goodness of fit *S* are based on *F*^2^, conventional *R*-factors *R* are based on *F*, with *F* set to zero for negative *F*^2^. The threshold expression of *F*^2^ > σ(*F*^2^) is used only for calculating *R*-factors(gt) *etc*. and is not relevant to the choice of reflections for refinement. *R*-factors based on *F*^2^ are statistically about twice as large as those based on *F*, and *R*- factors based on ALL data will be even larger.

### Fractional atomic coordinates and isotropic or equivalent isotropic displacement parameters (Å^2^)

**Table:**

	*x*	*y*	*z*	*U*_iso_*/*U*_eq_	
Si	0.18831 (6)	0.75264 (12)	0.35551 (8)	0.0440 (4)	
S	0.03335 (5)	0.23627 (11)	0.13817 (6)	0.0309 (3)	
O1	0.02908 (13)	0.1117 (3)	0.12092 (17)	0.0387 (8)	
O2	−0.01544 (11)	0.3124 (3)	0.08511 (16)	0.0360 (8)	
O3	0.18264 (12)	0.2966 (3)	0.15322 (18)	0.0394 (8)	
O4	0.08605 (14)	0.4847 (3)	0.03241 (17)	0.0401 (8)	
H4	0.0674	0.4249	0.0053	0.048*	
O5	0.15140 (15)	0.8445 (3)	0.27897 (18)	0.0514 (10)	
N	0.09151 (13)	0.2865 (3)	0.13766 (19)	0.0283 (9)	
C1	0.04472 (17)	0.2563 (4)	0.2366 (2)	0.0319 (11)	
C2	0.03227 (18)	0.3650 (4)	0.2570 (3)	0.0344 (12)	
H2B	0.0194	0.4288	0.2196	0.041*	
C3	0.0387 (2)	0.3797 (4)	0.3326 (3)	0.0408 (13)	
H3A	0.0301	0.4544	0.3466	0.049*	
C4	0.0573 (2)	0.2888 (5)	0.3882 (3)	0.0398 (13)	
C5	0.06901 (19)	0.1796 (5)	0.3667 (3)	0.0386 (13)	
H5A	0.0810	0.1155	0.4038	0.046*	
C6	0.06346 (17)	0.1633 (4)	0.2923 (3)	0.0330 (11)	
H6A	0.0724	0.0887	0.2786	0.040*	
C7	0.0632 (2)	0.3055 (5)	0.4703 (3)	0.0537 (15)	
H7A	0.0324	0.2595	0.4721	0.081*	
H7B	0.0584	0.3893	0.4783	0.081*	
H7C	0.1027	0.2787	0.5135	0.081*	
C8	0.14300 (19)	0.2100 (4)	0.1522 (3)	0.0355 (12)	
C9	0.1269 (2)	0.1295 (4)	0.0813 (3)	0.0439 (13)	
H9A	0.1074	0.1753	0.0306	0.066*	
H9B	0.0997	0.0680	0.0785	0.066*	
H9C	0.1632	0.0929	0.0886	0.066*	
C10	0.17202 (19)	0.1457 (4)	0.2342 (3)	0.0408 (13)	
H10A	0.1820	0.2028	0.2778	0.061*	
H10B	0.2084	0.1061	0.2443	0.061*	
H10C	0.1442	0.0870	0.2330	0.061*	
C11	0.17841 (18)	0.3992 (4)	0.1934 (3)	0.0406 (13)	
H11B	0.1911	0.4705	0.1766	0.049*	
H11C	0.2037	0.3906	0.2533	0.049*	
C12	0.11234 (17)	0.4078 (4)	0.1666 (3)	0.0290 (11)	
H12B	0.1086	0.4258	0.2147	0.035*	
C13	0.07738 (19)	0.4990 (4)	0.0990 (3)	0.0314 (11)	
H13A	0.0339	0.4915	0.0789	0.038*	
C14	0.0980 (2)	0.6178 (4)	0.1337 (3)	0.0372 (12)	
C15	0.1154 (2)	0.7111 (5)	0.1641 (3)	0.0435 (14)	
C16	0.1359 (3)	0.8307 (4)	0.1969 (3)	0.0614 (17)	
H16A	0.1710	0.8504	0.1929	0.074*	
H16B	0.1039	0.8877	0.1626	0.074*	
C17	0.2209 (2)	0.8472 (4)	0.4478 (3)	0.0461 (13)	
C18	0.2571 (2)	0.7734 (5)	0.5270 (3)	0.0722 (18)	
H18A	0.2747	0.8261	0.5744	0.108*	
H18B	0.2889	0.7306	0.5255	0.108*	
H18C	0.2306	0.7168	0.5312	0.108*	
C19	0.1699 (3)	0.9130 (6)	0.4479 (4)	0.090 (2)	
H19A	0.1862	0.9621	0.4970	0.135*	
H19B	0.1418	0.8558	0.4475	0.135*	
H19C	0.1492	0.9632	0.3994	0.135*	
C20	0.2636 (3)	0.9367 (5)	0.4439 (4)	0.089 (2)	
H20A	0.2766	0.9945	0.4879	0.133*	
H20B	0.2429	0.9775	0.3912	0.133*	
H20C	0.2984	0.8951	0.4502	0.133*	
C21	0.1340 (2)	0.6438 (5)	0.3553 (3)	0.0633 (17)	
H21A	0.1090	0.6837	0.3720	0.095*	
H21B	0.1560	0.5795	0.3935	0.095*	
H21C	0.1087	0.6114	0.3004	0.095*	
C22	0.2475 (2)	0.6744 (6)	0.3472 (3)	0.0757 (19)	
H22A	0.2699	0.7317	0.3354	0.114*	
H22B	0.2290	0.6161	0.3032	0.114*	
H22C	0.2747	0.6344	0.3988	0.114*	
S'	0.47434 (5)	0.22934 (11)	0.36956 (6)	0.0314 (3)	
Si'	0.32749 (6)	0.77784 (12)	0.12641 (8)	0.0407 (4)	
O1'	0.47552 (13)	0.1048 (3)	0.38495 (18)	0.0382 (8)	
O2'	0.52277 (11)	0.3035 (3)	0.42724 (16)	0.0363 (8)	
O3'	0.31902 (12)	0.2997 (3)	0.33146 (18)	0.0400 (8)	
O4'	0.41055 (14)	0.4856 (3)	0.45876 (18)	0.0404 (8)	
H4'	0.4219	0.4204	0.4830	0.048*	
O5'	0.33037 (14)	0.8161 (3)	0.21186 (18)	0.0499 (10)	
N'	0.41365 (13)	0.2818 (3)	0.36147 (18)	0.0270 (8)	
C1'	0.47066 (16)	0.2498 (4)	0.2766 (2)	0.0290 (10)	
C2'	0.48723 (19)	0.3566 (4)	0.2598 (3)	0.0375 (12)	
H2'A	0.5016	0.4178	0.2994	0.045*	
C3'	0.4831 (2)	0.3752 (5)	0.1858 (3)	0.0425 (13)	
H3'A	0.4945	0.4492	0.1750	0.051*	
C4'	0.46240 (19)	0.2872 (5)	0.1274 (3)	0.0364 (12)	
C5'	0.44717 (19)	0.1792 (4)	0.1453 (3)	0.0360 (12)	
H5'A	0.4342	0.1172	0.1064	0.043*	
C6'	0.45037 (17)	0.1595 (4)	0.2184 (3)	0.0332 (11)	
H6'A	0.4389	0.0854	0.2291	0.040*	
C7'	0.4592 (2)	0.3072 (5)	0.0479 (3)	0.0486 (14)	
H7'A	0.4213	0.2766	0.0031	0.073*	
H7'B	0.4923	0.2660	0.0487	0.073*	
H7'C	0.4619	0.3919	0.0401	0.073*	
C8'	0.35917 (18)	0.2103 (4)	0.3387 (3)	0.0349 (12)	
C9'	0.3689 (2)	0.1310 (4)	0.4080 (3)	0.0440 (13)	
H9'A	0.3824	0.1784	0.4572	0.066*	
H9'B	0.3992	0.0716	0.4183	0.066*	
H9'C	0.3315	0.0915	0.3933	0.066*	
C10'	0.33513 (19)	0.1429 (4)	0.2585 (3)	0.0416 (13)	
H10D	0.2975	0.1045	0.2441	0.062*	
H10E	0.3642	0.0830	0.2649	0.062*	
H10F	0.3284	0.1981	0.2153	0.062*	
C11'	0.32856 (19)	0.3992 (4)	0.2954 (3)	0.0403 (13)	
H11A	0.3144	0.4719	0.3086	0.048*	
H11D	0.3072	0.3904	0.2354	0.048*	
C12'	0.39582 (17)	0.4040 (4)	0.3316 (3)	0.0303 (11)	
H12'	0.4044	0.4206	0.2875	0.036*	
C13'	0.4279 (2)	0.4945 (4)	0.4004 (2)	0.0309 (11)	
H13'	0.4720	0.4833	0.4278	0.037*	
C14'	0.41146 (19)	0.6151 (5)	0.3636 (3)	0.0350 (12)	
C15'	0.3987 (2)	0.7067 (5)	0.3305 (3)	0.0413 (13)	
C16'	0.3825 (2)	0.8229 (4)	0.2900 (3)	0.0543 (16)	
H16C	0.3761	0.8795	0.3239	0.065*	
H16D	0.4158	0.8528	0.2848	0.065*	
C17'	0.2463 (2)	0.7598 (4)	0.0517 (3)	0.0496 (13)	
C18'	0.2355 (2)	0.7335 (6)	−0.0336 (3)	0.0703 (17)	
H18D	0.1930	0.7174	−0.0714	0.105*	
H18E	0.2590	0.6646	−0.0308	0.105*	
H18F	0.2475	0.8019	−0.0530	0.105*	
C19'	0.2225 (2)	0.6565 (5)	0.0798 (4)	0.0709 (18)	
H19D	0.1793	0.6491	0.0425	0.106*	
H19E	0.2311	0.6722	0.1351	0.106*	
H19F	0.2420	0.5830	0.0797	0.106*	
C20'	0.2138 (2)	0.8750 (5)	0.0482 (4)	0.0700 (19)	
H20D	0.2301	0.9405	0.0325	0.105*	
H20E	0.2194	0.8910	0.1019	0.105*	
H20F	0.1711	0.8667	0.0079	0.105*	
C21'	0.3582 (2)	0.8992 (5)	0.0935 (3)	0.0562 (15)	
H21D	0.3367	0.9725	0.0884	0.084*	
H21E	0.3533	0.8791	0.0408	0.084*	
H21F	0.4006	0.9096	0.1341	0.084*	
C22'	0.3710 (2)	0.6407 (4)	0.1417 (3)	0.0499 (15)	
H22D	0.4128	0.6541	0.1839	0.075*	
H22E	0.3683	0.6191	0.0905	0.075*	
H22F	0.3547	0.5767	0.1587	0.075*	

### Atomic displacement parameters (Å^2^)

**Table:**

	*U*^11^	*U*^22^	*U*^33^	*U*^12^	*U*^13^	*U*^23^
Si	0.0502 (8)	0.0307 (9)	0.0424 (7)	0.0001 (7)	0.0179 (6)	−0.0018 (7)
S	0.0358 (6)	0.0252 (7)	0.0308 (6)	−0.0035 (6)	0.0166 (5)	0.0012 (6)
O1	0.053 (2)	0.027 (2)	0.0371 (18)	−0.0117 (16)	0.0237 (16)	−0.0072 (15)
O2	0.0289 (16)	0.037 (2)	0.0368 (17)	0.0023 (15)	0.0131 (14)	0.0092 (16)
O3	0.0410 (18)	0.0301 (19)	0.0530 (19)	−0.0014 (16)	0.0286 (15)	−0.0008 (17)
O4	0.058 (2)	0.030 (2)	0.0303 (19)	−0.0085 (17)	0.0209 (17)	−0.0041 (15)
O5	0.078 (2)	0.029 (2)	0.037 (2)	−0.0034 (18)	0.0224 (18)	−0.0009 (15)
N	0.0334 (19)	0.022 (2)	0.0320 (19)	−0.0066 (17)	0.0192 (16)	−0.0029 (18)
C1	0.035 (2)	0.029 (3)	0.033 (2)	−0.003 (2)	0.019 (2)	0.003 (2)
C2	0.039 (3)	0.026 (3)	0.042 (3)	0.002 (2)	0.024 (2)	0.007 (2)
C3	0.054 (3)	0.028 (3)	0.048 (3)	0.005 (2)	0.031 (3)	0.000 (2)
C4	0.042 (3)	0.044 (3)	0.039 (3)	−0.003 (3)	0.024 (2)	0.001 (3)
C5	0.044 (3)	0.038 (3)	0.037 (3)	0.008 (3)	0.023 (2)	0.014 (3)
C6	0.040 (3)	0.024 (3)	0.035 (3)	0.005 (2)	0.020 (2)	0.007 (2)
C7	0.073 (4)	0.057 (4)	0.046 (3)	0.001 (3)	0.041 (3)	−0.003 (3)
C8	0.039 (3)	0.033 (3)	0.035 (3)	0.002 (2)	0.020 (2)	0.000 (2)
C9	0.056 (3)	0.036 (3)	0.046 (3)	0.008 (2)	0.031 (3)	−0.003 (3)
C10	0.044 (3)	0.032 (3)	0.044 (3)	0.004 (2)	0.021 (2)	0.004 (2)
C11	0.036 (3)	0.035 (3)	0.047 (3)	−0.005 (2)	0.019 (2)	0.002 (2)
C12	0.037 (3)	0.020 (3)	0.028 (2)	−0.007 (2)	0.015 (2)	−0.005 (2)
C13	0.035 (2)	0.020 (3)	0.035 (3)	−0.004 (2)	0.016 (2)	−0.001 (2)
C14	0.047 (3)	0.026 (3)	0.033 (3)	−0.001 (2)	0.018 (2)	0.002 (2)
C15	0.062 (3)	0.029 (3)	0.033 (3)	−0.004 (3)	0.020 (2)	0.003 (3)
C16	0.108 (5)	0.027 (3)	0.045 (3)	−0.017 (3)	0.037 (3)	−0.006 (3)
C17	0.049 (3)	0.040 (3)	0.039 (3)	−0.004 (3)	0.016 (2)	0.002 (2)
C18	0.085 (4)	0.055 (4)	0.045 (3)	0.001 (3)	0.012 (3)	0.006 (3)
C19	0.117 (5)	0.086 (5)	0.074 (4)	0.041 (4)	0.055 (4)	−0.003 (4)
C20	0.100 (5)	0.059 (5)	0.083 (5)	−0.048 (4)	0.031 (4)	−0.015 (4)
C21	0.063 (3)	0.047 (4)	0.077 (4)	−0.016 (3)	0.034 (3)	0.004 (3)
C22	0.069 (4)	0.073 (5)	0.074 (4)	0.018 (4)	0.030 (3)	−0.017 (4)
S'	0.0359 (6)	0.0280 (8)	0.0318 (6)	0.0042 (6)	0.0187 (6)	−0.0004 (6)
Si'	0.0483 (7)	0.0317 (9)	0.0401 (7)	0.0042 (7)	0.0218 (6)	0.0005 (6)
O1'	0.051 (2)	0.027 (2)	0.0393 (18)	0.0105 (16)	0.0256 (16)	0.0076 (15)
O2'	0.0321 (16)	0.042 (2)	0.0323 (16)	0.0030 (15)	0.0147 (13)	−0.0079 (16)
O3'	0.0355 (17)	0.038 (2)	0.0504 (19)	0.0021 (16)	0.0249 (15)	−0.0024 (17)
O4'	0.059 (2)	0.030 (2)	0.0347 (19)	0.0080 (17)	0.0263 (17)	0.0047 (16)
O5'	0.067 (2)	0.045 (2)	0.0360 (18)	0.0150 (18)	0.0250 (17)	0.0099 (17)
N'	0.0312 (18)	0.021 (2)	0.0297 (18)	0.0037 (17)	0.0166 (15)	0.0032 (17)
C1'	0.027 (2)	0.030 (3)	0.030 (2)	−0.002 (2)	0.0154 (18)	−0.004 (2)
C2'	0.046 (3)	0.032 (3)	0.042 (3)	−0.006 (2)	0.028 (2)	−0.009 (2)
C3'	0.060 (3)	0.031 (3)	0.050 (3)	−0.011 (3)	0.038 (3)	−0.004 (3)
C4'	0.036 (3)	0.044 (3)	0.032 (2)	−0.002 (2)	0.020 (2)	−0.001 (3)
C5'	0.044 (3)	0.031 (3)	0.033 (3)	−0.003 (2)	0.020 (2)	−0.007 (2)
C6'	0.036 (2)	0.024 (3)	0.039 (3)	−0.005 (2)	0.019 (2)	−0.004 (2)
C7'	0.057 (3)	0.057 (4)	0.043 (3)	−0.001 (3)	0.034 (2)	0.001 (3)
C8'	0.034 (2)	0.032 (3)	0.043 (3)	0.001 (2)	0.023 (2)	0.001 (2)
C9'	0.053 (3)	0.040 (3)	0.049 (3)	−0.010 (2)	0.033 (3)	0.003 (3)
C10'	0.046 (3)	0.035 (3)	0.044 (3)	−0.007 (2)	0.023 (2)	−0.008 (2)
C11'	0.043 (3)	0.029 (3)	0.044 (3)	0.005 (2)	0.020 (2)	−0.001 (2)
C12'	0.033 (2)	0.026 (3)	0.031 (3)	0.008 (2)	0.016 (2)	−0.001 (2)
C13'	0.044 (3)	0.022 (3)	0.026 (3)	0.005 (2)	0.018 (2)	0.005 (2)
C14'	0.036 (3)	0.036 (3)	0.033 (3)	0.000 (2)	0.018 (2)	−0.006 (3)
C15'	0.056 (3)	0.031 (3)	0.035 (3)	−0.002 (2)	0.022 (2)	−0.001 (2)
C16'	0.083 (4)	0.025 (3)	0.039 (3)	−0.003 (3)	0.020 (3)	−0.002 (2)
C17'	0.057 (3)	0.039 (3)	0.058 (3)	−0.003 (3)	0.033 (2)	0.003 (3)
C18'	0.066 (3)	0.091 (5)	0.046 (3)	−0.020 (4)	0.023 (3)	−0.008 (4)
C19'	0.075 (4)	0.055 (4)	0.101 (5)	−0.026 (3)	0.058 (4)	−0.011 (4)
C20'	0.053 (3)	0.061 (5)	0.090 (5)	0.010 (3)	0.033 (3)	0.006 (3)
C21'	0.059 (3)	0.041 (3)	0.077 (4)	−0.007 (3)	0.042 (3)	0.000 (3)
C22'	0.059 (3)	0.034 (3)	0.058 (3)	0.016 (3)	0.031 (3)	0.003 (3)

### Geometric parameters (Å, °)

**Table:**

Si—O5	1.644 (3)	S'—O1'	1.439 (3)
Si—C17	1.856 (5)	S'—O2'	1.444 (3)
Si—C22	1.868 (5)	S'—N'	1.632 (3)
Si—C21	1.887 (5)	S'—C1'	1.754 (4)
S—O2	1.437 (3)	Si'—O5'	1.662 (3)
S—O1	1.442 (3)	Si'—C22'	1.859 (5)
S—N	1.636 (3)	Si'—C17'	1.861 (4)
S—C1	1.769 (4)	Si'—C21'	1.863 (5)
O3—C8	1.424 (5)	O3'—C11'	1.413 (6)
O3—C11	1.432 (6)	O3'—C8'	1.416 (5)
O4—C13	1.421 (5)	O4'—C13'	1.418 (5)
O4—H4	0.8400	O4'—H4'	0.8400
O5—C16	1.417 (6)	O5'—C16'	1.412 (5)
N—C12	1.478 (5)	N'—C12'	1.479 (5)
N—C8	1.506 (6)	N'—C8'	1.499 (5)
C1—C2	1.383 (6)	C1'—C2'	1.380 (6)
C1—C6	1.397 (6)	C1'—C6'	1.400 (6)
C2—C3	1.381 (6)	C2'—C3'	1.385 (7)
C2—H2B	0.9500	C2'—H2'A	0.9500
C3—C4	1.378 (7)	C3'—C4'	1.384 (7)
C3—H3A	0.9500	C3'—H3'A	0.9500
C4—C5	1.389 (7)	C4'—C5'	1.386 (6)
C4—C7	1.514 (6)	C4'—C7'	1.502 (6)
C5—C6	1.373 (6)	C5'—C6'	1.380 (6)
C5—H5A	0.9500	C5'—H5'A	0.9500
C6—H6A	0.9500	C6'—H6'A	0.9500
C7—H7A	0.9800	C7'—H7'A	0.9800
C7—H7B	0.9800	C7'—H7'B	0.9800
C7—H7C	0.9800	C7'—H7'C	0.9800
C8—C9	1.506 (6)	C8'—C9'	1.514 (6)
C8—C10	1.532 (6)	C8'—C10'	1.531 (6)
C9—H9A	0.9800	C9'—H9'A	0.9800
C9—H9B	0.9800	C9'—H9'B	0.9800
C9—H9C	0.9800	C9'—H9'C	0.9800
C10—H10A	0.9800	C10'—H10D	0.9800
C10—H10B	0.9800	C10'—H10E	0.9800
C10—H10C	0.9800	C10'—H10F	0.9800
C11—C12	1.536 (6)	C11'—C12'	1.527 (6)
C11—H11B	0.9900	C11'—H11A	0.9900
C11—H11C	0.9900	C11'—H11D	0.9900
C12—C13	1.535 (6)	C12'—C13'	1.533 (6)
C12—H12B	1.0000	C12'—H12'	1.0000
C13—C14	1.476 (7)	C13'—C14'	1.495 (7)
C13—H13A	1.0000	C13'—H13'	1.0000
C14—C15	1.179 (6)	C14'—C15'	1.171 (6)
C15—C16	1.474 (7)	C15'—C16'	1.477 (7)
C16—H16A	0.9900	C16'—H16C	0.9900
C16—H16B	0.9900	C16'—H16D	0.9900
C17—C19	1.533 (7)	C17'—C18'	1.541 (7)
C17—C20	1.543 (7)	C17'—C20'	1.543 (7)
C17—C18	1.555 (6)	C17'—C19'	1.551 (7)
C18—H18A	0.9800	C18'—H18D	0.9800
C18—H18B	0.9800	C18'—H18E	0.9800
C18—H18C	0.9800	C18'—H18F	0.9800
C19—H19A	0.9800	C19'—H19D	0.9800
C19—H19B	0.9800	C19'—H19E	0.9800
C19—H19C	0.9800	C19'—H19F	0.9800
C20—H20A	0.9800	C20'—H20D	0.9800
C20—H20B	0.9800	C20'—H20E	0.9800
C20—H20C	0.9800	C20'—H20F	0.9800
C21—H21A	0.9800	C21'—H21D	0.9800
C21—H21B	0.9800	C21'—H21E	0.9800
C21—H21C	0.9800	C21'—H21F	0.9800
C22—H22A	0.9800	C22'—H22D	0.9800
C22—H22B	0.9800	C22'—H22E	0.9800
C22—H22C	0.9800	C22'—H22F	0.9800
			
O5—Si—C17	104.6 (2)	O1'—S'—O2'	119.81 (19)
O5—Si—C22	110.7 (3)	O1'—S'—N'	107.00 (19)
C17—Si—C22	111.5 (2)	O2'—S'—N'	106.73 (18)
O5—Si—C21	108.6 (2)	O1'—S'—C1'	108.8 (2)
C17—Si—C21	110.6 (3)	O2'—S'—C1'	105.3 (2)
C22—Si—C21	110.7 (3)	N'—S'—C1'	108.81 (18)
O2—S—O1	119.40 (18)	O5'—Si'—C22'	110.7 (2)
O2—S—N	106.14 (19)	O5'—Si'—C17'	103.6 (2)
O1—S—N	107.5 (2)	C22'—Si'—C17'	113.2 (2)
O2—S—C1	105.8 (2)	O5'—Si'—C21'	109.7 (2)
O1—S—C1	108.8 (2)	C22'—Si'—C21'	109.4 (2)
N—S—C1	108.89 (18)	C17'—Si'—C21'	110.1 (2)
C8—O3—C11	107.8 (3)	C11'—O3'—C8'	107.8 (3)
C13—O4—H4	109.5	C13'—O4'—H4'	109.5
C16—O5—Si	128.3 (3)	C16'—O5'—Si'	125.7 (3)
C12—N—C8	110.2 (3)	C12'—N'—C8'	109.4 (3)
C12—N—S	119.0 (3)	C12'—N'—S'	118.3 (3)
C8—N—S	123.4 (3)	C8'—N'—S'	124.6 (3)
C2—C1—C6	119.7 (4)	C2'—C1'—C6'	119.4 (4)
C2—C1—S	119.0 (3)	C2'—C1'—S'	119.8 (3)
C6—C1—S	121.2 (4)	C6'—C1'—S'	120.9 (4)
C3—C2—C1	119.2 (4)	C1'—C2'—C3'	120.5 (4)
C3—C2—H2B	120.4	C1'—C2'—H2'A	119.7
C1—C2—H2B	120.4	C3'—C2'—H2'A	119.7
C4—C3—C2	121.7 (5)	C4'—C3'—C2'	120.7 (5)
C4—C3—H3A	119.2	C4'—C3'—H3'A	119.7
C2—C3—H3A	119.2	C2'—C3'—H3'A	119.7
C3—C4—C5	118.6 (5)	C3'—C4'—C5'	118.5 (4)
C3—C4—C7	121.1 (5)	C3'—C4'—C7'	120.4 (5)
C5—C4—C7	120.3 (4)	C5'—C4'—C7'	121.0 (4)
C6—C5—C4	120.7 (5)	C6'—C5'—C4'	121.5 (5)
C6—C5—H5A	119.7	C6'—C5'—H5'A	119.2
C4—C5—H5A	119.7	C4'—C5'—H5'A	119.2
C5—C6—C1	120.0 (5)	C5'—C6'—C1'	119.3 (5)
C5—C6—H6A	120.0	C5'—C6'—H6'A	120.3
C1—C6—H6A	120.0	C1'—C6'—H6'A	120.3
C4—C7—H7A	109.5	C4'—C7'—H7'A	109.5
C4—C7—H7B	109.5	C4'—C7'—H7'B	109.5
H7A—C7—H7B	109.5	H7'A—C7'—H7'B	109.5
C4—C7—H7C	109.5	C4'—C7'—H7'C	109.5
H7A—C7—H7C	109.5	H7'A—C7'—H7'C	109.5
H7B—C7—H7C	109.5	H7'B—C7'—H7'C	109.5
O3—C8—N	100.7 (4)	O3'—C8'—N'	101.0 (4)
O3—C8—C9	106.8 (4)	O3'—C8'—C9'	106.7 (4)
N—C8—C9	112.3 (3)	N'—C8'—C9'	111.5 (3)
O3—C8—C10	110.2 (3)	O3'—C8'—C10'	111.2 (3)
N—C8—C10	112.5 (4)	N'—C8'—C10'	113.2 (4)
C9—C8—C10	113.4 (4)	C9'—C8'—C10'	112.4 (4)
C8—C9—H9A	109.5	C8'—C9'—H9'A	109.5
C8—C9—H9B	109.5	C8'—C9'—H9'B	109.5
H9A—C9—H9B	109.5	H9'A—C9'—H9'B	109.5
C8—C9—H9C	109.5	C8'—C9'—H9'C	109.5
H9A—C9—H9C	109.5	H9'A—C9'—H9'C	109.5
H9B—C9—H9C	109.5	H9'B—C9'—H9'C	109.5
C8—C10—H10A	109.5	C8'—C10'—H10D	109.5
C8—C10—H10B	109.5	C8'—C10'—H10E	109.5
H10A—C10—H10B	109.5	H10D—C10'—H10E	109.5
C8—C10—H10C	109.5	C8'—C10'—H10F	109.5
H10A—C10—H10C	109.5	H10D—C10'—H10F	109.5
H10B—C10—H10C	109.5	H10E—C10'—H10F	109.5
O3—C11—C12	105.3 (3)	O3'—C11'—C12'	105.3 (4)
O3—C11—H11B	110.7	O3'—C11'—H11A	110.7
C12—C11—H11B	110.7	C12'—C11'—H11A	110.7
O3—C11—H11C	110.7	O3'—C11'—H11D	110.7
C12—C11—H11C	110.7	C12'—C11'—H11D	110.7
H11B—C11—H11C	108.8	H11A—C11'—H11D	108.8
N—C12—C13	111.5 (3)	N'—C12'—C11'	101.6 (4)
N—C12—C11	101.5 (4)	N'—C12'—C13'	111.8 (3)
C13—C12—C11	113.6 (4)	C11'—C12'—C13'	113.7 (4)
N—C12—H12B	110.0	N'—C12'—H12'	109.8
C13—C12—H12B	110.0	C11'—C12'—H12'	109.8
C11—C12—H12B	110.0	C13'—C12'—H12'	109.8
O4—C13—C14	108.5 (4)	O4'—C13'—C14'	107.9 (4)
O4—C13—C12	112.3 (4)	O4'—C13'—C12'	112.0 (4)
C14—C13—C12	108.3 (3)	C14'—C13'—C12'	108.1 (3)
O4—C13—H13A	109.2	O4'—C13'—H13'	109.6
C14—C13—H13A	109.2	C14'—C13'—H13'	109.6
C12—C13—H13A	109.2	C12'—C13'—H13'	109.6
C15—C14—C13	177.7 (5)	C15'—C14'—C13'	175.6 (5)
C14—C15—C16	176.4 (5)	C14'—C15'—C16'	179.0 (6)
O5—C16—C15	114.0 (4)	O5'—C16'—C15'	111.4 (4)
O5—C16—H16A	108.8	O5'—C16'—H16C	109.3
C15—C16—H16A	108.8	C15'—C16'—H16C	109.3
O5—C16—H16B	108.8	O5'—C16'—H16D	109.3
C15—C16—H16B	108.8	C15'—C16'—H16D	109.3
H16A—C16—H16B	107.7	H16C—C16'—H16D	108.0
C19—C17—C20	109.7 (5)	C18'—C17'—C20'	109.0 (4)
C19—C17—C18	110.5 (5)	C18'—C17'—C19'	109.9 (4)
C20—C17—C18	108.1 (4)	C20'—C17'—C19'	109.8 (4)
C19—C17—Si	108.3 (3)	C18'—C17'—Si'	110.1 (3)
C20—C17—Si	108.6 (4)	C20'—C17'—Si'	109.1 (3)
C18—C17—Si	111.5 (4)	C19'—C17'—Si'	108.8 (3)
C17—C18—H18A	109.5	C17'—C18'—H18D	109.5
C17—C18—H18B	109.5	C17'—C18'—H18E	109.5
H18A—C18—H18B	109.5	H18D—C18'—H18E	109.5
C17—C18—H18C	109.5	C17'—C18'—H18F	109.5
H18A—C18—H18C	109.5	H18D—C18'—H18F	109.5
H18B—C18—H18C	109.5	H18E—C18'—H18F	109.5
C17—C19—H19A	109.5	C17'—C19'—H19D	109.5
C17—C19—H19B	109.5	C17'—C19'—H19E	109.5
H19A—C19—H19B	109.5	H19D—C19'—H19E	109.5
C17—C19—H19C	109.5	C17'—C19'—H19F	109.5
H19A—C19—H19C	109.5	H19D—C19'—H19F	109.5
H19B—C19—H19C	109.5	H19E—C19'—H19F	109.5
C17—C20—H20A	109.5	C17'—C20'—H20D	109.5
C17—C20—H20B	109.5	C17'—C20'—H20E	109.5
H20A—C20—H20B	109.5	H20D—C20'—H20E	109.5
C17—C20—H20C	109.5	C17'—C20'—H20F	109.5
H20A—C20—H20C	109.5	H20D—C20'—H20F	109.5
H20B—C20—H20C	109.5	H20E—C20'—H20F	109.5
Si—C21—H21A	109.5	Si'—C21'—H21D	109.5
Si—C21—H21B	109.5	Si'—C21'—H21E	109.5
H21A—C21—H21B	109.5	H21D—C21'—H21E	109.5
Si—C21—H21C	109.5	Si'—C21'—H21F	109.5
H21A—C21—H21C	109.5	H21D—C21'—H21F	109.5
H21B—C21—H21C	109.5	H21E—C21'—H21F	109.5
Si—C22—H22A	109.5	Si'—C22'—H22D	109.5
Si—C22—H22B	109.5	Si'—C22'—H22E	109.5
H22A—C22—H22B	109.5	H22D—C22'—H22E	109.5
Si—C22—H22C	109.5	Si'—C22'—H22F	109.5
H22A—C22—H22C	109.5	H22D—C22'—H22F	109.5
H22B—C22—H22C	109.5	H22E—C22'—H22F	109.5
			
C17—Si—O5—C16	155.8 (4)	C17'—Si'—O5'—C16'	169.9 (4)
C22—Si—O5—C16	35.6 (5)	C21'—Si'—O5'—C16'	−72.6 (4)
C21—Si—O5—C16	−86.1 (5)	O1'—S'—N'—C12'	−169.6 (3)
O2—S—N—C12	61.0 (3)	O2'—S'—N'—C12'	61.0 (3)
O1—S—N—C12	−170.2 (3)	C1'—S'—N'—C12'	−52.2 (3)
C1—S—N—C12	−52.6 (3)	O1'—S'—N'—C8'	−23.5 (4)
O2—S—N—C8	−151.9 (3)	O2'—S'—N'—C8'	−152.9 (3)
O1—S—N—C8	−23.1 (3)	C1'—S'—N'—C8'	94.0 (4)
C1—S—N—C8	94.6 (3)	O1'—S'—C1'—C2'	−160.1 (3)
O2—S—C1—C2	−31.0 (4)	O2'—S'—C1'—C2'	−30.4 (4)
O1—S—C1—C2	−160.4 (3)	N'—S'—C1'—C2'	83.7 (4)
N—S—C1—C2	82.7 (4)	O1'—S'—C1'—C6'	20.7 (4)
O2—S—C1—C6	146.2 (3)	O2'—S'—C1'—C6'	150.4 (3)
O1—S—C1—C6	16.7 (4)	N'—S'—C1'—C6'	−95.5 (4)
N—S—C1—C6	−100.1 (4)	C6'—C1'—C2'—C3'	0.9 (7)
C6—C1—C2—C3	0.1 (6)	S'—C1'—C2'—C3'	−178.3 (4)
S—C1—C2—C3	177.3 (4)	C1'—C2'—C3'—C4'	−0.2 (7)
C1—C2—C3—C4	0.0 (7)	C2'—C3'—C4'—C5'	−1.3 (7)
C2—C3—C4—C5	−0.8 (7)	C2'—C3'—C4'—C7'	−178.8 (4)
C2—C3—C4—C7	−179.0 (4)	C3'—C4'—C5'—C6'	2.1 (7)
C3—C4—C5—C6	1.5 (7)	C7'—C4'—C5'—C6'	179.5 (4)
C7—C4—C5—C6	179.6 (4)	C4'—C5'—C6'—C1'	−1.4 (7)
C4—C5—C6—C1	−1.3 (7)	C2'—C1'—C6'—C5'	−0.2 (7)
C2—C1—C6—C5	0.5 (6)	S'—C1'—C6'—C5'	179.0 (3)
S—C1—C6—C5	−176.7 (3)	C11'—O3'—C8'—N'	38.2 (4)
C11—O3—C8—N	37.8 (4)	C11'—O3'—C8'—C9'	154.9 (4)
C11—O3—C8—C9	155.2 (4)	C11'—O3'—C8'—C10'	−82.2 (4)
C11—O3—C8—C10	−81.2 (4)	C12'—N'—C8'—O3'	−24.8 (4)
C12—N—C8—O3	−25.0 (4)	S'—N'—C8'—O3'	−173.5 (3)
S—N—C8—O3	−174.6 (3)	C12'—N'—C8'—C9'	−137.9 (4)
C12—N—C8—C9	−138.3 (4)	S'—N'—C8'—C9'	73.4 (5)
S—N—C8—C9	72.1 (5)	C12'—N'—C8'—C10'	94.3 (4)
C12—N—C8—C10	92.3 (4)	S'—N'—C8'—C10'	−54.4 (5)
S—N—C8—C10	−57.3 (4)	C8'—O3'—C11'—C12'	−37.7 (5)
C8—O3—C11—C12	−37.1 (5)	C8'—N'—C12'—C11'	3.4 (4)
C8—N—C12—C13	125.1 (4)	S'—N'—C12'—C11'	154.4 (3)
S—N—C12—C13	−83.8 (4)	C8'—N'—C12'—C13'	125.0 (4)
C8—N—C12—C11	3.8 (4)	S'—N'—C12'—C13'	−84.0 (4)
S—N—C12—C11	155.0 (3)	O3'—C11'—C12'—N'	19.6 (5)
O3—C11—C12—N	19.0 (4)	O3'—C11'—C12'—C13'	−100.6 (4)
O3—C11—C12—C13	−100.8 (4)	N'—C12'—C13'—O4'	−66.3 (5)
N—C12—C13—O4	−63.6 (5)	C11'—C12'—C13'—O4'	48.0 (5)
C11—C12—C13—O4	50.3 (5)	N'—C12'—C13'—C14'	175.0 (4)
N—C12—C13—C14	176.6 (4)	C11'—C12'—C13'—C14'	−70.7 (5)
C11—C12—C13—C14	−69.5 (5)	Si'—O5'—C16'—C15'	−84.5 (5)
O5—Si—C17—C19	57.4 (4)	O5'—Si'—C17'—C18'	175.5 (4)
C22—Si—C17—C19	177.1 (4)	C22'—Si'—C17'—C18'	−64.5 (4)
C21—Si—C17—C19	−59.3 (4)	C21'—Si'—C17'—C18'	58.2 (5)
O5—Si—C17—C20	−61.7 (4)	O5'—Si'—C17'—C20'	55.9 (4)
C22—Si—C17—C20	58.0 (4)	C22'—Si'—C17'—C20'	175.8 (4)
C21—Si—C17—C20	−178.5 (4)	C21'—Si'—C17'—C20'	−61.4 (4)
O5—Si—C17—C18	179.2 (4)	O5'—Si'—C17'—C19'	−63.9 (4)
C22—Si—C17—C18	−61.1 (5)	C22'—Si'—C17'—C19'	56.0 (4)
C21—Si—C17—C18	62.5 (4)	C21'—Si'—C17'—C19'	178.8 (4)
C22'—Si'—O5'—C16'	48.3 (4)		

### Hydrogen-bond geometry (Å, °)

**Table:**

*D*—H···*A*	*D*—H	H···*A*	*D*···*A*	*D*—H···*A*
O4—H4···O2^i^	0.84	2.01	2.828 (4)	164
O4'—H4'···O2'^ii^	0.84	2.06	2.851 (4)	157

Symmetry codes: (i) −*x*, *y*, −*z*; (ii) −*x*+1, *y*, −*z*+1.
